# A Multi-User Transradial Functional-Test Socket for Validation of New Myoelectric Prosthetic Control Strategies

**DOI:** 10.3389/fnbot.2022.872791

**Published:** 2022-06-17

**Authors:** Taylor C. Hansen, Abigail R. Citterman, Eric S. Stone, Troy N. Tully, Christopher M. Baschuk, Christopher C. Duncan, Jacob A. George

**Affiliations:** ^1^Department of Biomedical Engineering, University of Utah, Salt Lake City, UT, United States; ^2^Handspring, Salt Lake City, UT, United States; ^3^Department of Physical Medicine and Rehabilitation, University of Utah, Salt Lake City, UT, United States; ^4^Departments of Electrical and Computer Engineering and Mechanical Engineering, University of Utah, Salt Lake City, UT, United States

**Keywords:** prosthetics, socket design, myoelectric, fabrication techniques, transradial

## Abstract

The validation of myoelectric prosthetic control strategies for individuals experiencing upper-limb loss is hindered by the time and cost affiliated with traditional custom-fabricated sockets. Consequently, researchers often rely upon virtual reality or robotic arms to validate novel control strategies, which limits end-user involvement. Prosthetists fabricate diagnostic check sockets to assess and refine socket fit, but these clinical techniques are not readily available to researchers and are not intended to assess functionality for control strategies. Here we present a multi-user, low-cost, transradial, functional-test socket for short-term research use that can be custom-fit and donned rapidly, used in conjunction with various electromyography configurations, and adapted for use with various residual limbs and terminal devices. In this study, participants with upper-limb amputation completed functional tasks in physical and virtual environments both with and without the socket, and they reported on their perceived comfort level over time. The functional-test socket was fabricated prior to participants' arrival, iteratively fitted by the researchers within 10 mins, and donned in under 1 min (excluding electrode placement, which will vary for different use cases). It accommodated multiple individuals and terminal devices and had a total cost of materials under $10 USD. Across all participants, the socket did not significantly impede functional task performance or reduce the electromyography signal-to-noise ratio. The socket was rated as comfortable enough for at least 2 h of use, though it was expectedly perceived as less comfortable than a clinically-prescribed daily-use socket. The development of this multi-user, transradial, functional-test socket constitutes an important step toward increased end-user participation in advanced myoelectric prosthetic research. The socket design has been open-sourced and is available for other researchers.

## Introduction

Despite significant advances in the multiarticulate capabilities of upper-limb myoelectric prostheses in recent decades, up to half of people with upper-limb amputation continue to abandon these advanced myoelectric prostheses in favor of more traditional body-powered devices or no prosthesis at all (Biddiss and Chau, 2007b; Østlie et al., 2012; Salminger et al., 2020). Users cite many contributors to their dissatisfaction, among them unreliable prosthetic control (Biddiss and Chau, 2007a; Espinosa and Nathan-Roberts, 2019). The most recent advances in myoelectric control are not yet commercially available and remain limited to research laboratories. Improving user acceptance of myoelectric prostheses necessitates adequate user involvement in the development process, including in a research setting. Unfortunately, recruiting a satisfactory number of research participants to assess novel myoelectric control strategies is impeded by the high fabrication cost and low electrode count of traditional, individualized myoelectric sockets. Alternatives to embedding electrodes into sockets, such as intrafascicular electrodes or targeted muscle reinnervation, are surgically invasive and even more costly to implement (Engdahl et al., 2015). Despite considerable interest in myoelectric control from individuals with upper-limb amputation (Engdahl et al., 2017), user involvement in research studies is often limited to just one or a few individuals with upper-limb amputation (Hebert and Lewicke, 2012; Jiang et al., 2014a; Amsuess et al., 2015) often working in virtual reality environments (Kumar and Todorov, 2015; Kluger et al., 2019; Teh and Hargrove, 2020) or with a robotic arm mounted apart from the user (Ninu et al., 2014; Chadwell et al., 2016; Zhuang et al., 2019). Other studies validate only with healthy participants (Englehart and Hudgins, 2003; Hahne et al., 2014; Jiang et al., 2014a,b; Vidovic et al., 2016; Parajuli et al., 2019) or offline analyses in which there is no active human-in-the-loop involvement (Ortiz-Catalan et al., 2015; Geng et al., 2017).

One approach to increasing the number of studies that validate results with individuals with upper-limb amputation is to reduce the cost per participant by using an adjustable socket (Infinite-Socket^*TM*^-TF; Schofield et al., 2019). An adjustable myoelectric socket could accommodate dynamic changes in the residual limb that would otherwise require casting a new socket. Additionally, an adjustable myoelectric socket could be used with multiple individuals and multiple terminal devices. Recent work toward reducing the cost of custom, individualized sockets has focused on 3D printing, embedded gel liners, and expandable foams (Buis et al., 2017; Reissman et al., 2018; Hallworth et al., 2020; Ismail et al., 2020; Miller et al., 2020; Olsen et al., 2021). These sockets have the added advantage of not requiring a prosthetist for the fabrication and fitting processes. Notwithstanding, these approaches still require a lengthy fabrication process for each person, involving protracted laboratory visits before the participant can begin using the socket. While sockets that are both customizable and affordable have been explored, they have yet to be adapted for myoelectric prosthesis use (Thomas et al., 2015). It should be noted that prosthetists traditionally fabricate diagnostic check sockets to quickly assess and refine socket fit. However, these clinical check sockets are not readily available to researchers and are not intended to assess functionality for myoelectric control strategies. Clinical check sockets are used primarily to validate the length and comfort of the socket, and they do not accommodate electrodes for electromyography.

To address these needs, we developed a multi-user, 3D-printed, transradial, functional-test socket for validation of new myoelectric control strategies in research settings, hereafter referred to as simply “functional-test socket.” By “functional-test” we mean that the socket allows for functional testing of prosthetic control. Researchers can fabricate the components prior to the participants' arrival, allowing participants to don and use the customized socket within minutes. We explored its comfort and functionality with a high-count surface electromyography (sEMG) control system. Subsequent use by other participants does not typically necessitate reprinting of parts, as the 3D-printed material can be reshaped. The development of this socket constitutes an important step toward expanding the involvement of individuals with transradial amputation in state-of-the-art myoelectric-control research.

## Materials and Methods

### Device Development

#### Criteria

##### Accessibility

To expand participant involvement, the socket must be easily and rapidly manufactured. Following fabrication, researchers must be able to quickly customize the fit and don the socket with minimal to no training. Many researchers are not trained prosthetists, making a straightforward design critical for ensuring timely implementation.

Additionally, the socket must be manufactured at a low cost, utilizing common manufacturing and printing materials, to ensure accessibility. Conventional transradial sockets intended for long-term use range from $800 to $3,000 USD (Limb Prostheses (Conventional) Product Manual, 2012; Frossard et al., 2017), with recently developed low-cost alternatives ranging from $100 to $200 USD—the cost of which may still be prohibitively high—making the desired cost under $100 USD (Alkhatib et al., 2019; Ismail et al., 2020; Miller et al., 2020). A device intended for short-term research could feasibly be even more economical still.

##### Adaptability

The implementation of this socket for myoelectric research purposes must accommodate disparate data acquisition (DAQ) methods, thereby allowing for the exploration of various control systems (Roche et al., 2014; Geethanjali, 2016). Additionally, a proper fit is essential for socket comfort and functionality (Østlie et al., 2012; Salminger et al., 2020). The socket must therefore be customizable while maintaining cost- and time efficiency. The design must allow for variability in residual limbs and terminal devices to further expand user involvement. Specifically, the design should be able to readily accommodate sensitive spots on the residual limb (e.g., due to neuromas, bone protrusions, or wounds).

##### Durability

With the variance in weight of transradial prostheses, the socket must be able to support 2.3 kg to accommodate commercially available devices (i-Limb® Ultra; TASKA^*TM*^; Michelangelo; bebionic). This support requirement should account for both vertical load suspension (i.e., tension) as well as horizontal load suspension (i.e., torque). The mechanical properties of a 3D-printed device are dependent on material selection and print orientation (Song et al., 2017; Alkhatib et al., 2019; Maroti et al., 2019). To optimize the strength of a socket without neglecting comfort, considerations must be made to the computer-aided design in addition to the material selection. A socket must be conducive to activities of daily living (ADLs) and therefore durable enough to withstand loads beyond that of the terminal devices without deflection or discomfort. Commercially available myoelectric hands can lift a maximum weight ranging from 20 kg (e.g., TASKA, Michelangelo) up to 90 kg (e.g., i-Limb) (TASKA^*TM*^; i-Limb® Ultra; Michelangelo). The ideal socket would be durable enough to not impose new limits with the use of such prostheses.

#### Design

**Figure 1:**
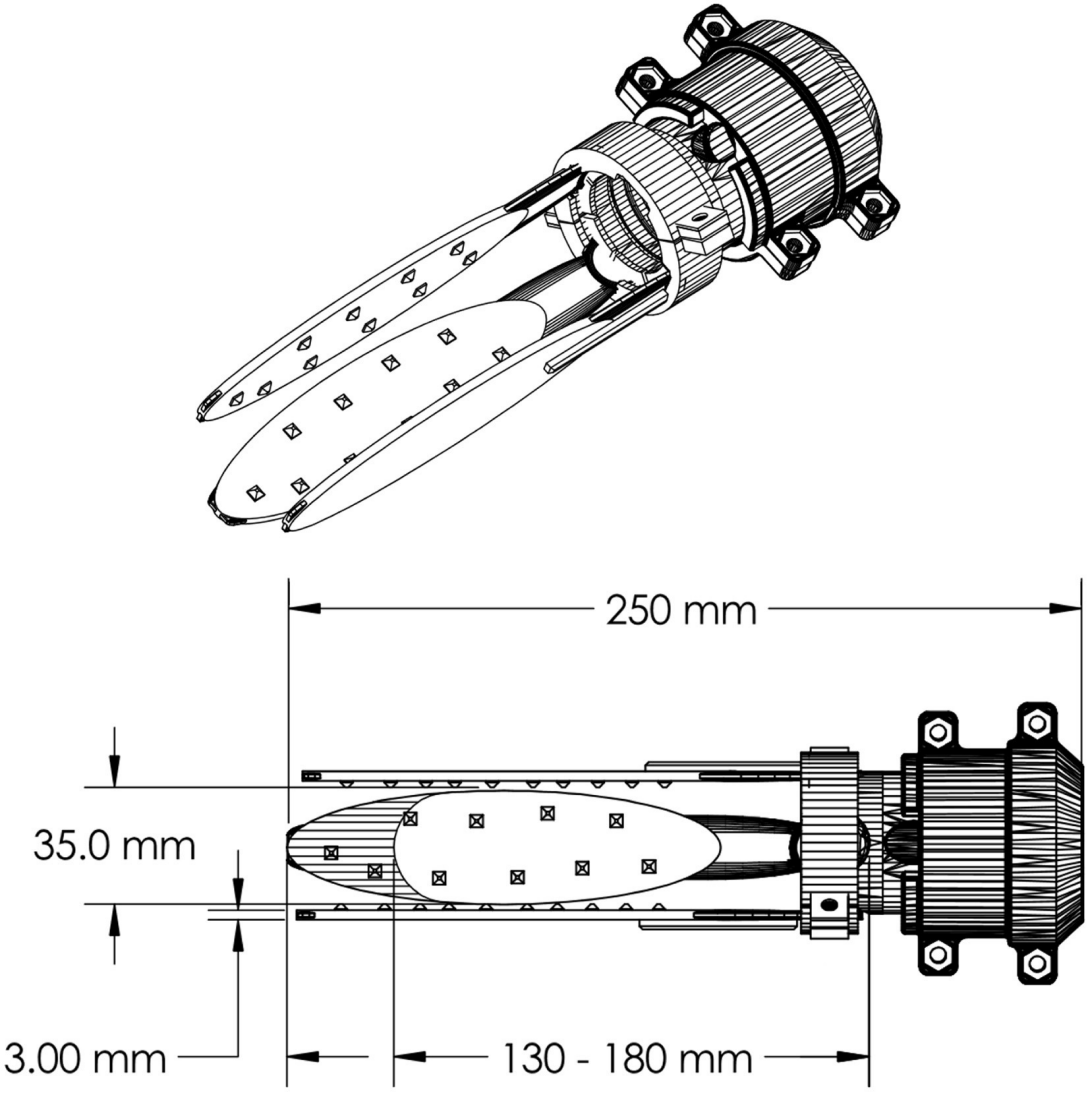
Functional-test socket design with important dimensions of the socket indicated. Only three of the four flexible struts are depicted. The socket can accommodate variable residual limb lengths by trimming the struts to the desired length. The struts can also be heated and reshaped to adapt to the residual limb shape of a given user. The struts are nested inside of a collet which in turn houses a custom adapter for a terminal device. The adapter for the TASKA hand is pictured here. The socket design has been open-sourced and is available for other researchers at https://github.com/utahneurorobotics/u-of-u-functional-test-socket.

**Figure 2:**
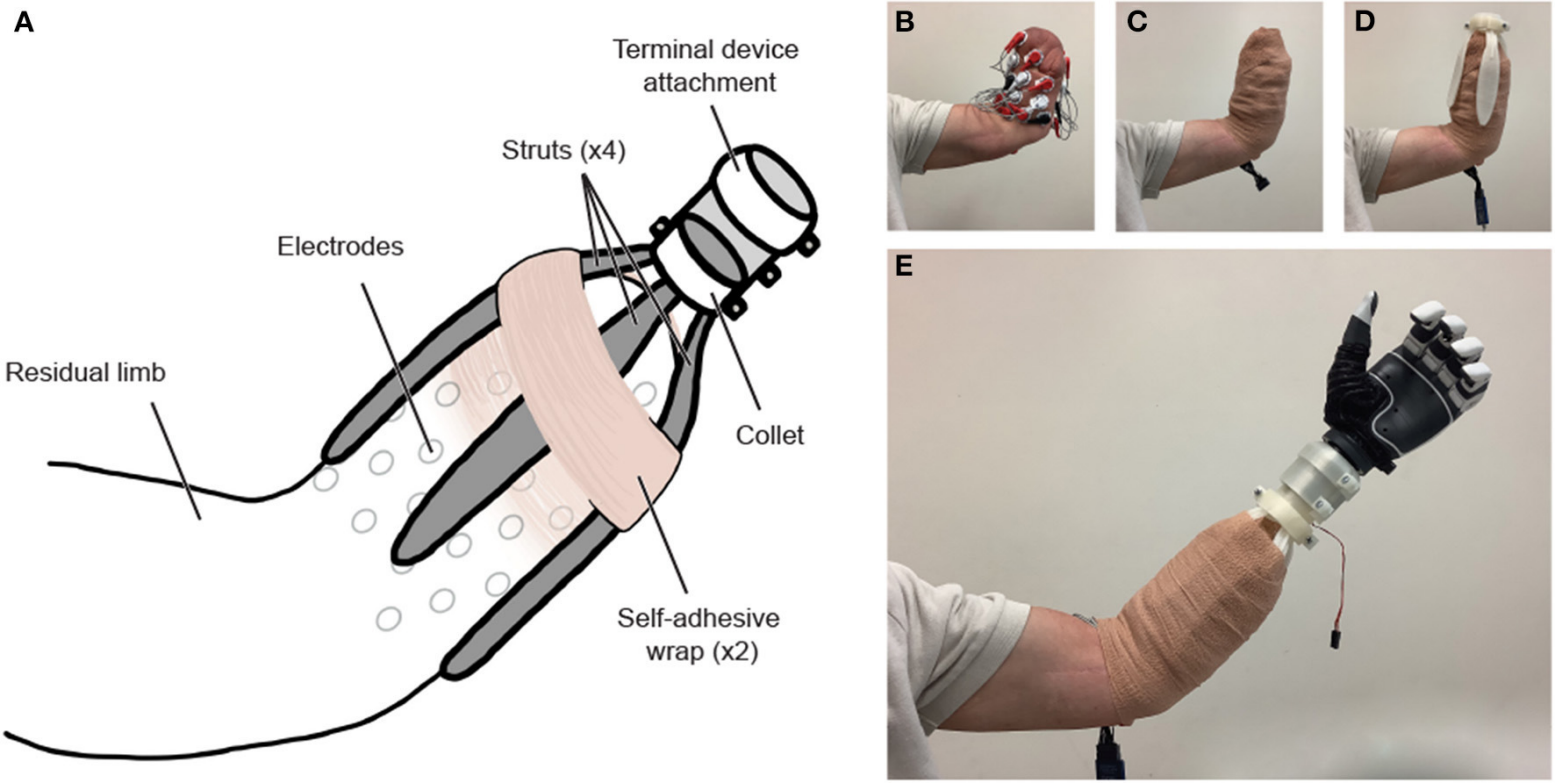
**(A)** Functional-test socket overview. **(B)** The residual limb is outfitted with sEMG electrodes and **(C)** wrapped securely in a disposable adhesive bandage. **(D)** After being heated, molded, and cut to the desired length, the custom-fit struts are attached to the collet, and the socket is donned. **(E)** A second layer of adhesive bandage is wrapped around the limb and socket system, and the socket is fit with a myoelectric prosthesis, pictured above with a TASKA hand. The modular terminal device attachment is conducive to use with a variety of terminal devices. The 3D-printed components can be removed, reshaped, and reused with a subsequent individual without requiring reprinting.

We designed our functional-test socket to optimize accessibility and cost, accommodate a range of DAQ methods, residual limbs, and prostheses, and ensure functionality and comfort with high-count electromyography. The 3D-printed socket consists of four customizable struts that attach to a collet, which in turn connects to a custom terminal device attachment that varies for each unique terminal device (Figure 1). When donning the socket, a layer of self-adhesive wrap between the skin and the socket offers grip, and a second layer around the socket secures the fit (Figure 2). The socket design has been open-sourced and is available for other researchers, along with additional details regarding socket assembly, at https://github.com/utahneurorobotics/u-of-u-functional-test-socket.

##### Accessibility

The 3D-printable design of the socket improves accessibility, enabling researchers without prosthetist expertise to complete both the fabrication and fitting processes. The socket is printed prior to the participant's arrival. Upon arrival, the custom-fitting process can be completed within 10 mins, and the socket can be donned in under 1 min (Table 1). The donning time does not include the time needed to place electrodes on the residual limb, as this time will vary depending on the data acquisition system selected by the researchers.

**Table 1:** Time approximation, by process.

**Fabrication (3D printing)**	**Fitting (molding struts)**	**Donning**	**Doffing**
6 h, 30 mins	10 mins	<1 min^*^	<1 min

* *Excluding electrode placement, which varies depending on selected data acquisition system*.

The total cost of materials of one socket is ~$8.00 USD (Table 2), excluding other printer costs such as maintenance. The choice of myoelectric control system is at the researchers' discretion and is thus not included in this estimate. The components (i.e., 3D-printed components, hardware, self-adhesive wrap, and memory foam) are widely available materials. The accessibility of this design is conducive to greater participant involvement and allows for those with transradial amputations to begin validating advanced myoelectric prostheses promptly.

**Table 2:** Cost analysis (listed in USD).

**3D-printed components (i.e., filament)**	**Hardware (i.e., nuts and bolts)**	**Self-adhesive wrap**	**Memory foam**	**Total**
$3.50	$1.00	$3.00	$0.50	**$8.00**

##### Adaptability

Our socket is designed to be versatile and is compatible with a range of control methods, residual limbs, and terminal devices. The custom fit of the socket grants access to the skin for various means of control (e.g., sEMG, magnetomyography, sonomyography, tactile myography) (Kim and Colgate, 2012; Heidari et al., 2018; Calado et al., 2019; Dhawan et al., 2019; Connan et al., 2020). The 3D-printed struts can then be molded around a selected DAQ method without affecting overall socket fit or comfort. We note that our own tests were limited to using sEMG as the DAQ method. The polylactic acid (PLA) filament we selected allows the struts to be heated in a hot water bath or with a heat gun and quickly molded to the unique presentation of the participant's residual limb. Importantly, the same components can be reheated and remolded for use with a subsequent participant without needing to be reprinted, further reducing the cost per participant. This reheating and reshaping process has been shown to not significantly degrade the mechanical properties of the PLA (Chalgham et al., 2021).

The adaptability of the socket improves the overall participant experience by accommodating limb-volume fluctuations and avoiding any painful sites (e.g., bone protrusions, neuromas, wounds) by orienting the struts around the forearm according to the user's preference. The struts can be cut to accommodate residual-limb length during the fitting process. It has been tested with residual limb lengths between 15 and 20 cm (Table 3). The secure custom fit allows for ADLs to be performed as with a standard socket, albeit for a finite time in a research setting. The design is adaptable to other open-source connectors that can be printed along with the socket to accommodate a variety of commercially available prostheses (e.g., DEKA and TASKA hands; available at https://github.com/utahneurorobotics/u-of-u-functional-test-socket). Such adaptability is also conducive to improved hand orientation, as the default position of the prosthesis can be adjusted by rotating the terminal device attachment within the collet (Figure 2A).

**Table 3:** Participant demographics.

**Participant**	**Sex and age**	**Amputation description**	**BMI**	**Elbow to end of residual limb (cm)**	**Residual limb circumference (cm)**	**Myoelectric prosthesis experience?**
P1	Male aged 65	Bilateral traumatic transradial (3 years)	29.5	20	27	Some
P2	Male aged 44	Bilateral traumatic transradial (10 years)	24.4	15	26	Some
P3	Male aged 63	Left traumatic transradial (20 years)	25.8	19.5	26.5	Extensive

##### Durability

The struts are printed flat to ensure structural stability (Song et al., 2017; Maroti et al., 2019) and take their contoured shape only in the custom-fitting process. The material selection of PLA offers greater toughness and higher break elongation, break load, and break strength when compared to other thermoplastic filaments commonly utilized in 3D printing, such as ABS and HIPS (Martin and Avérous, 2001; Kumar et al., 2018). The struts are designed to minimize deflection and slipping; a thicker layer of filament along the top of each strut for reinforcement supports weight and maintains structural integrity, and surface texture along the bottom creates grip with the self-adhesive bandage. Furthermore, the placement of the four struts around the limb in conjunction with the increased surface area of the wide, paddle-like struts helps to distribute the pressure more evenly throughout the socket. Such weight distribution is key to supporting loads beyond that of the terminal devices and accommodating ADLs. The weight of the socket is ~150 g, which is at the low end of the 100- to 420-g range for traditional sockets and low-cost alternatives (Lenka and Choudhury, 2011; Ismail et al., 2020). Incorporating a layer of memory foam beneath the greatest load-bearing strut further increases comfort. Altogether, these design considerations ensure comfort while promoting greater maximum load and overall durability.

### Testing

#### Mechanical Testing

Before participant recruitment, we tested the mechanical capabilities of the functional-test socket. To facilitate this testing, a plaster residual-limb replica was fabricated to which the socket could be attached according to the process depicted in Figure 2. Importantly, during mechanical testing, we opted to exclude the electrode placement in the donning process to demonstrate that the reliability of the socket to adhere to the limb was not dependent on additional friction from the electrodes. We also note that, for mechanical testing, the struts were heated and molded into shape a single time. After affixing the socket to the plaster residuum, two modes of load suspension were evaluated: vertical and horizontal. To ensure that the testing was not dependent on the capabilities of a given terminal device, loads were suspended from a custom 3D-printed loop attached to the wrist portion of the socket in place of a functional terminal device.

The two modes of load suspension tested the limit use cases of the socket. Vertical load suspension is most prone to cause the socket to slip off the residual limb; it is also the orientation in which the terminal device is most likely to dislodge from the 3D-printed quick-disconnect collet. During vertical load suspension, masses up to a total of 8 kg were incrementally and statically hung from the loop for several minutes, after which the amount, if any, by which the socket had slipped downward was measured. We chose this mass as it is roughly twice that of a gallon of water, which has been used previously to explore loading for ADLs (Drew et al., 2017). Additionally, at the maximal load, the socket was raised and lowered rapidly to simulate a dynamic load condition such as going down stairs.

The second mode for loading was in a horizontal orientation. Horizontal load suspension is most likely to induce socket fracturing, either at the connection to the terminal device or along the dorsal struts placed superficially to the residual limb. To test these potential failure points, the socket was oriented horizontally, and masses up to a total of 8 kg were hung vertically from the terminal loop for several minutes. Finally, the degree, if any, by which the socket had deflected downward due to the strain from the masses was measured with a goniometer, and any fractures in the socket components were noted. Each of the two loading modes (vertical and horizontal) was evaluated once.

#### Signal Acquisition and Control

Three participants (P1-P3) with transradial amputations were recruited to assess the functional-test socket (Table 3). The study was approved by the Institutional Review Board of the University of Utah, and written informed consent was obtained from each participant. To illustrate the socket's compatibility with high degree-of-freedom myoelectric prostheses, 32 single-ended sEMG signals were collected from the participants' residual limbs *via* a Grapevine Neural Interface Processor system (Ripple Neuro LLC, Salt Lake City, UT, USA). Although we opted for this high-count system in the present work, the functional-test socket design could potentially work with any number of sEMG acquisition systems, including a standard two-site control system.

To provide control of a prosthesis, sEMG signals were recorded while participants mimicked movements of a virtual prosthetic hand (Davoodi et al., 2007; Paskett et al., 2021). The movements consisted of 10 full, simultaneous flexions (grasps) of all digits and 10 full, simultaneous extensions of all digits. The resulting data was used to train a modified Kalman filter (mKF), which allowed for continuous, proportional control of a prosthetic hand (George et al., 2020a). This procedure was carried out both with the functional-test socket donned and with it doffed.

#### Functional Testing

We aimed to demonstrate that the functional-test socket was both comfortable and functional. To that end, each participant reported on their perceived level of socket comfort at three time points in the experimental session: shortly after donning, halfway through the experiment, and just prior to doffing (~2 h after donning). At each time point, comfort was self-reported using a 0–10 Likert scale, where 0 was the least comfortable and 10 was the most comfortable (Hanspal et al., 2003). The minimum detectable change for this score is 2.7 points (90% confidence interval) (Hafner et al., 2016). Participants also performed various functional tasks both with and without the socket. Where possible, the tasks were counterbalanced to mitigate order effects and the potential confound of fatigue. For each participant, the functional tasks included a signal-to-noise ratio (SNR) calculation, a target-touching task, and a modified box and blocks test (BBT). Participants first completed an SNR calculation and five trials of the target-touching task without the socket. Next, with the socket donned, the participants did a second SNR calculation, 10 target-touching task trials, five trials of the modified BBT, and a third SNR calculation. The socket was then removed, and participants performed a fourth SNR calculation and five trials of the target-touching task. Each functional task is now described in further detail.

#### Signal-to-Noise Ratio

Signal quality was compared both with and without the socket. sEMG was recorded while participants performed four different movement sets: 1) isolated flexion and extension of individual digits for 10 s, 2) alternating flexion (grasping) and extension of all digits for 10 s, 3) free-form wrist movements in all three axes for 10 s, and 4) a 10 s rest period (Wendelken, 2018; George et al., 2020c). Participants were instructed to perform movements 1–3 at maximal effort. The SNR for each of the 32 surface electrodes during each movement set (1–3) was then calculated by dividing the mean absolute value (MAV) during the movement by the MAV during the rest period (George et al., 2020b). Each participant completed this task twice with the socket donned and twice with the socket doffed.

#### Target-Touching Task

Performance with and without the socket was evaluated *via* a target-touching task, which required participants to move and hold a virtual prosthetic hand in target positions for as long as possible during 7-s trials (Davoodi et al., 2007; Page et al., 2018). Control of the virtual hand was afforded *via* the mKF described above. During the task, the target positions were at either a 50% grasp or a 50% extension of all digits, and each target had a ±10% error tolerance as indicated by a color-coded sphere. Participants attempted 10 trials of each movement type (grasp and extend all) both with and without the socket.

#### Modified Box and Blocks Test

The functional-test socket was fitted with a TASKA prosthetic hand (TASKA Prosthetics, Christchurch, New Zealand) so that participants could complete the modified BBT (Hebert and Lewicke, 2012; Hebert et al., 2014). In this task, participants must transfer 16 blocks arranged in a grid from one compartment of a box to the other within 60 s. If a participant moved all blocks within the time limit, the number of transferred blocks was extrapolated to the full 60 s. Each participant attempted the modified BBT a total of five times.

### Statistical Analysis

Across all performance metrics, we tested if wearing the functional-test socket impacted both within-participant performance and group mean performance to interpret our results in the context of the larger patient population. Deviations from normality in the data were checked with the Anderson-Darling test, in which the null hypothesis was that the data came from a normal distribution. We failed to reject this null hypothesis at the 0.05 significance level for the data in each metric and thus used parametric inferential statistics to report the results. The significance level for all subsequent statistical tests was 0.05 unless otherwise stated.

We performed paired *t*-tests to test the null hypotheses that the mean SNRs were the same with and without the functional-test socket for each movement type. If the *t*-test resulted in a non-significant *p*-value, we failed to reject these null hypotheses. However, failure to reject these null hypotheses does not confirm that the SNR is functionally the same between the two cases (Lakens, 2017). To explicitly test if the two conditions were not statistically different, we also performed two one-sided tests for equivalence (TOST) to determine the minimum interval for which the SNR was statistically equivalent (Rogers et al., 1993).

Performance metrics from the target-touching task included: 1) root-mean-squared error (RMSE) of the prosthesis position compared to the targets (Dantas et al., 2021) and 2) percentage time in the target region. For each metric, paired *t*-tests were run to compare the no-socket and socket performance. Again, if a *t*-test resulted in a non-significant *p*-value, we performed a TOST to determine the equivalence window.

For the modified BBT, the number of blocks transferred was compared to that of other myoelectric prosthesis users from the literature who completed the modified BBT (Paskett et al., 2018; George et al., 2019) or the original BBT (Salminger et al., 2019), for a total of two comparisons per group. Since each comparison group had a different number of trials and participants, we used two-sample *t*-tests to test for differences between conditions. Significance was established for *p*-values ≤ 0.025 to account for multiple comparisons using the Bonferroni correction. Non-significance resulted in a subsequent TOST to identify the equivalence window between the functional-test socket's performance and the reported literature values.

## Results

### Mechanical Testing Demonstrates Socket Reliability in Expected Use Cases

The functional-test socket was subjected to mechanical testing in both vertical and horizontal orientations using a plaster replica of a residual limb. During vertical load suspension, a maximal load of 8 kg produced no measurable slipping of the socket from the limb. Similarly, no slipping was detected in the dynamic condition wherein the socket bearing a maximal 8 kg load was quickly raised up and down. In the horizontal load mode, the socket was again loaded with up to 8 kg of suspended masses to detect vertical deflection and any breakpoints at the connection to the terminal device or the load-bearing struts. At maximal load, <1° of vertical deflection was noted in the connection point between the dorsal-most strut and the collet for the terminal device. It was apparent, however, that this deflection was elastic in nature as the socket quickly reverted to its original form once the load was removed. No visible fractures were apparent in the socket components following this horizontal load condition.

### Signal-to-Noise Ratio Not Affected by Socket in Aggregate but Varied for Each Participant Individually

**Figure 3:**
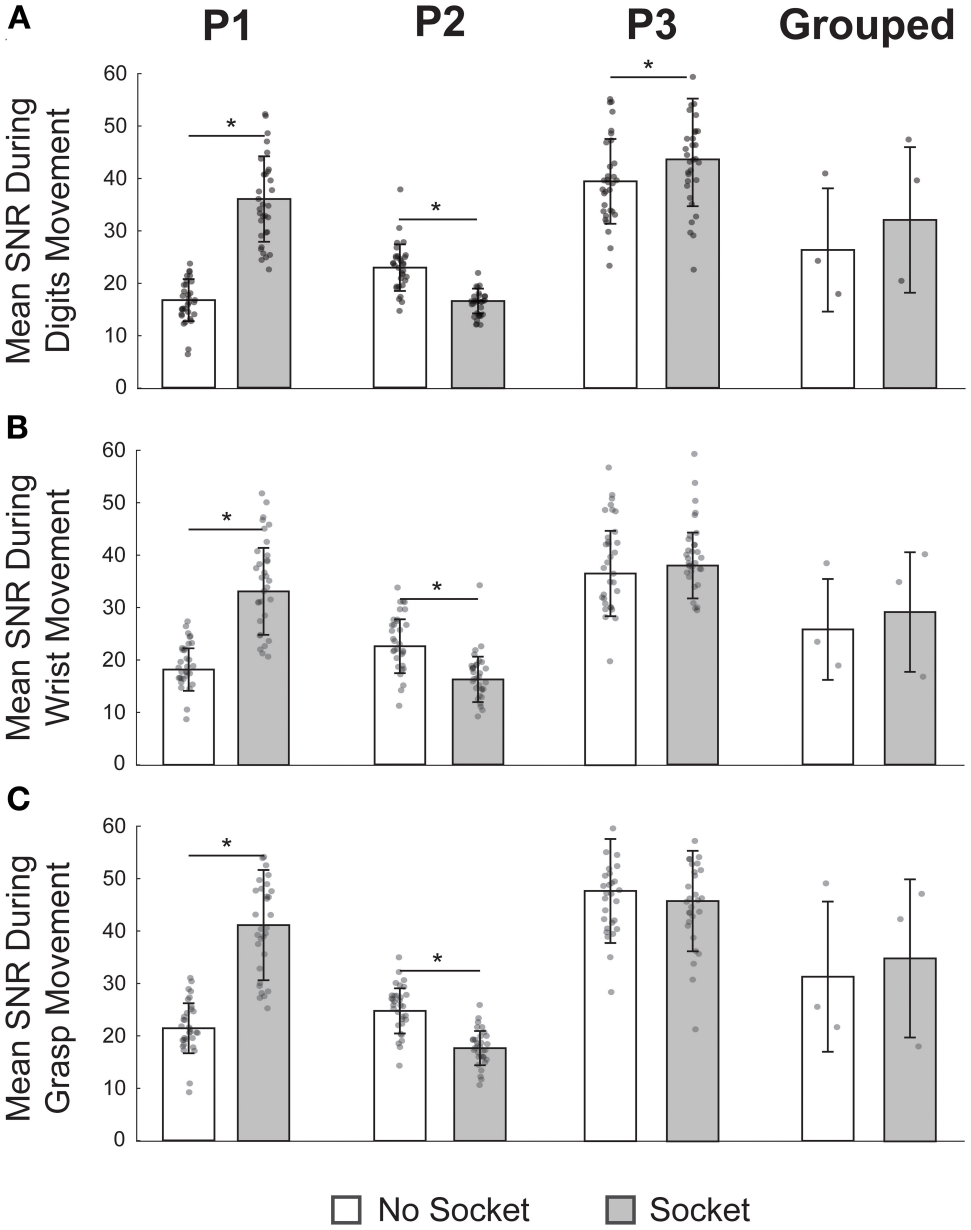
Signal-to-noise ratio (SNR) was not affected by the functional-test socket in aggregate but varied for each participant individually. **(A)** Mean SNR during movement of individual digits was significantly different at the individual level when wearing the socket but not so for the grouped case. **(B)** During wrist movements, SNR was significantly different for P1 and P2 but not so for P3 or in the grouped case. **(C)** Once again, SNR varied significantly for P1 and P2 during grasping but was statistically equivalent for P3 and in the grouped case. *p-value < 0.05, paired *t*-tests; *N* = 32 for each P1, P2, P3 bar; *N* = 3 for each grouped bar; individual data points are overlayed on each bar.

To test the impact of the socket on the SNR, we compared capabilities both with and without the functional-test socket during three different movement types. We found that, in aggregate (*N* = 3), performance (mean ± standard deviation) without and with the socket, respectively, was comparable during individual digit movements (26.3 ± 11.8 vs. 32.1 ± 13.9), grasping (32.0 ± 14.6 vs. 35.6 ± 15.4), and wrist movements (26.8 ± 10.0 vs. 30.2 ± 11.9) (Figure 3, far-right column; *p*'s > 0.05, paired *t*-tests). To determine the 90% confidence intervals of equivalence between the two conditions, we performed TOST for each movement type. We found that SNR with the socket was equivalent to no socket within −11.5 and +23.0 SNR for individual digit movements, within −16.5 and +23.8 SNR for grasping, and within −11.3 and +18.2 SNR for wrist movements (all *p*'s < 0.05, TOST). Interpreted another way, SNR with the socket was statistically equivalent to the SNR without the socket within 36% for individual digit movements (100 × 11.5/32.1), within 46% for grasping (100 × 16.5/35.6), and within 37% for wrist movements (100 × 11.3/30.2).

Given the low sample size (*N* = 3) of the aggregate SNRs, we also quantified SNR impact for each participant individually (Figure 3, columns 1–3, *N* = 32 per bar). For P1, the mean SNR with the socket was significantly higher than the SNR without the socket for all three movement types (*p*'s < 0.05, paired *t*-tests). In contrast, for P2, the mean SNR with the socket was significantly lower than the SNR without the socket (*p*'s < 0.05, paired *t*-tests). For P3, SNR was statistically greater with the socket for the individual digit movements (*p* = 0.03, paired *t*-test) but was not statistically different for the other two movement types (*p*'s > 0.05, paired *t*-tests). For the two movement types for P3 that were not statistically different, we again used a TOST procedure to determine the minimum statistical equivalence window. For grasping, the 90% confidence interval of equivalence was −6.0 to 2.2 SNR; for wrist movements, it was −1.7 to 4.5 SNR. In other words, for P3, the grasping SNR with the socket was statistically no worse than 13% (100 × 6.0/46.8) of the grasping SNR without the socket, and the wrist SNR with the socket was statistically no worse than 4% (100 × 1.7/39.4) of the wrist SNR without the socket.

### Performance During Target-Touching Task Not Impeded by Socket

**Figure 4:**
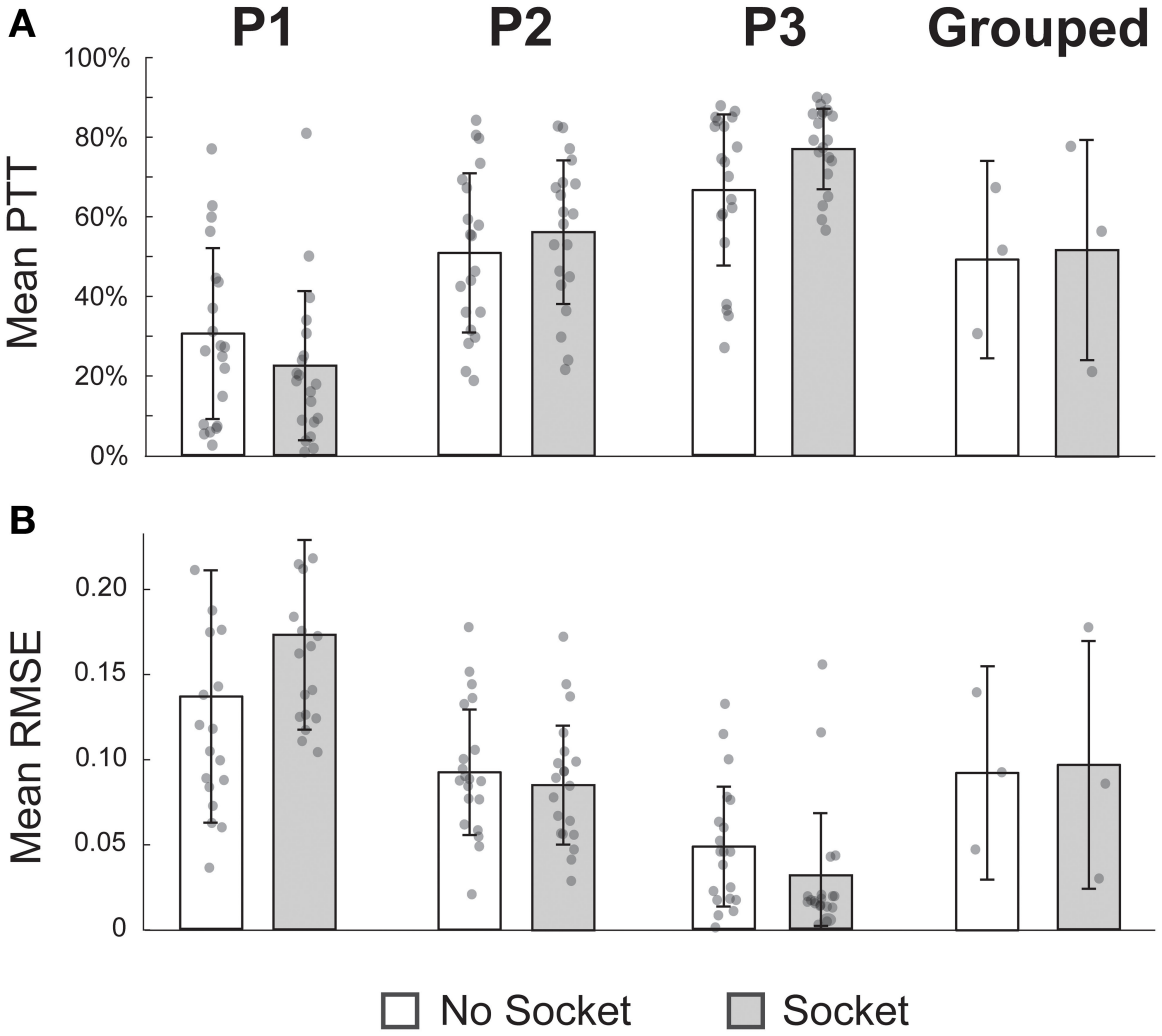
Performance during the target-touching task was not impeded by the socket. **(A)** Mean percent time in the target region (PTT) was not significantly different while wearing the socket for individual participants nor in the grouped case. **(B)** Similarly, mean root-mean-squared error (RMSE) was not significantly different when using the socket across the four comparisons. All *p*-values > 0.05, paired *t*-tests; *N* = 20 for each P1, P2, P3 bar; *N* = 3 for each grouped bar; individual data points are overlayed on each bar.

Across both performance metrics, there was no statistical difference between target-touching trials with or without the functional-test socket (Figure 4). When considering grouped performance (*N* = 3), trials without the socket were in the target region 50.2 ± 20.5% of the time, and trials with the socket were on target 52.8 ± 15.9% of the time (*p* = 0.68, Figure 4A, far-right column). Individual performance (*N* = 20 per bar) also showed comparable values between conditions. Percentage times in target (PTT) without and with the socket, respectively, were 31.1 ± 21.9% vs. 22.9 ± 19.1% for P1 (*p* = 0.29), 51.7 ± 20.4% vs. 57.2 ± 18.4% for P2 (*p* = 0.34), and 67.8 ± 19.3 vs. 78.4 ± 10.3% for P3 (*p* = 0.07).

The RMSE was also not significantly different between the two conditions. In the grouped case (*N* = 3), the mean RMSE for trials without the socket was 0.09 ± 0.05, compared to 0.09 ± 0.04 for trials with the socket (*p* = 0.83, Figure 4B, far-right column). Once again, individual performance (*N* = 20 per bar) was not significantly different if the socket was worn. RMSE values without and with the socket, respectively, were 0.14 ± 0.08 vs. 0.18 ± 0.06 for P1 (*p* = 0.06), 0.09 ± 0.04 vs. 0.09 ± 0.04 for P2 (*p* = 0.51), and 0.05 ± 0.04 vs. 0.03 ± 0.04 for P3 (*p* = 0.12).

TOST analyses were performed to determine equivalence windows between experimental conditions. For the PTT metric, the 90% confidence intervals of equivalence were −18.9 to 2.5%, −4.6 to 15.6%, 2.6 to 18.7%, and −29.2 to 34.5% for P1, P2, P3, and grouped, respectively (all *p*'s < 0.05, TOST). That is to say, the PTT with the socket was no worse than 83% (100 × 18.9/22.9) of the PTT without the socket for P1, no worse than 8% (100 × 4.6/57.2) for P2, no worse than 3% (100 × 2.6/78.4) for P3, and no worse than 55% (100 × 29.2/52.8) for the grouped case. Analogous tests were done for the RMSE metric, and the 90% confidence intervals were 0.00 to 0.07, −0.03 to 0.01, −0.04 to 0.00, and −0.08 to 0.09 for P1, P2, P3, and grouped, respectively (all *p*'s < 0.05, TOST). In other words, the RMSE with the socket was no worse than 1% (100 × 0.002/0.18) of the RMSE without the socket for P1, no worse than 33% (100 × 0.03/0.09) for P2, no worse than 133% (100 × 0.04/0.03) for P3, and no worse than 80% (100 × 0.08/0.10) for the grouped case.

### Modified Box and Blocks Test Performance Similar to Literature Values

**Figure 5:**
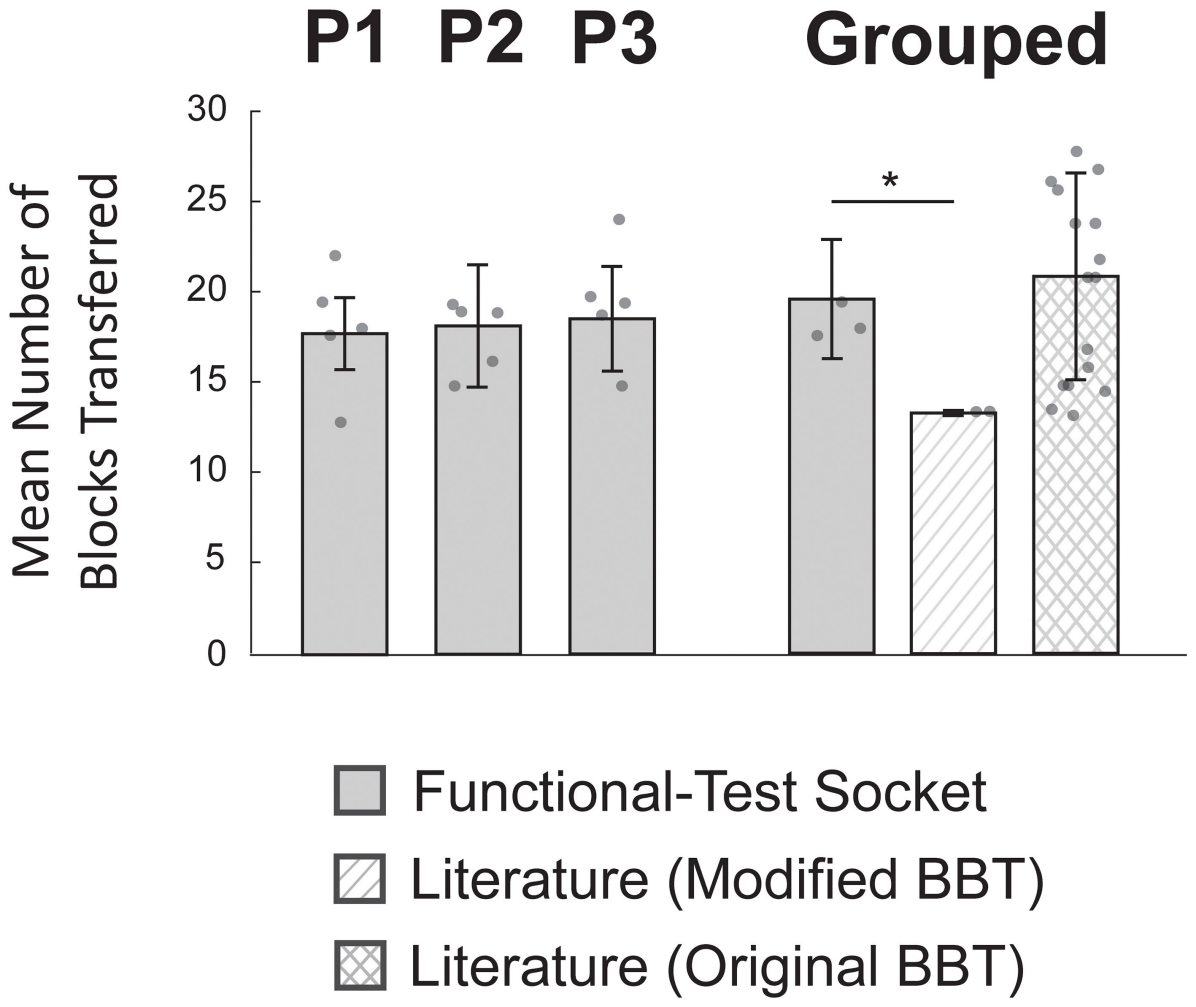
Modified box and blocks test (BBT) performance was similar to literature values. Individual participants (P1-P3) moved similar numbers of blocks across trials (*N* = 5 per bar). Individual performance was not statistically evaluated as the inter-subject variation was relatively small. In the grouped case, performance with the functional-test socket (*N* = 3) was not statistically different from values reported with the original BBT (*N* = 17). However, the grouped functional-test socket results did differ significantly compared to reported values for the modified BBT (*N* = 2). *p-value < 0.025, two-sample *t*-test corrected for multiple comparisons; individual data points are overlayed on each bar.

The average number of blocks transferred by the three participants with the functional-test socket was not reduced compared to values reported for myoelectric prosthesis users in the literature (Paskett et al., 2018; George et al., 2019; Salminger et al., 2019) (Figure 5). Participants using the functional-test socket (*N* = 3) transferred 19 ± 3 blocks in 60 s compared to literature values of 13 ± 0 blocks for the modified BBT (*N* = 2; Paskett et al., 2018; George et al., 2019) and 21 ± 6 blocks for the original BBT (*N* = 17; Salminger et al., 2019). The functional-test socket performance differed significantly from the literature values for the modified BBT but was not statistically different from the original BBT values (*p* = 0.005, *p* = 0.49, respectively).

Given the similarity between the grouped functional-test socket performance and the reported values for the original BBT, a TOST was performed to determine the equivalence window. The 90% confidence interval was found to be −8 to 3 blocks. In other words, the grouped performance with the functional-test socket was no worse than 43% (100 × 7.9/18.5) of the original BBT literature values. At the individual level (*N* = 5 per bar), P1 moved 18 ± 2 blocks, P2 moved 18 ± 3 blocks, and P3 moved 20 ± 3 blocks (Figure 5). Individual performance was not statistically evaluated as the inter-subject variation was relatively small.

### Comfort Remained Adequate During Experimental Sessions but Was Less Than a Traditional Socket and Imperceptibly Decreased Over Time for Most Participants

We asked each participant to rate the level of comfort of the functional-test socket at various time points throughout their visit. These scores were compared to the perceived comfort of their most-used traditional socket. Traditional socket scores were reported by participants to be 8.8 ± 1.3 on a 0–10 Likert scale. In comparison, shortly after donning, the functional-test socket scored 6.7 ± 1.2. Partway through the experiment, comfort scores were 6.5 ± 1.5. By the end of the experiment, comfort was reported as 5.7 ± 2.1. The mean difference of 1.0 in comfort scores is below the 2.7-point minimum detectable change (90% confidence interval) of this metric (Hafner et al., 2016). Of note, P3 reported no degradation in comfort scores for the functional-test socket for the duration of his visit and maintained comparable comfort levels compared to his traditional socket (8.0 vs. 9.0, respectively). The participant with the lowest reported comfort score for the functional-test socket, 4.0, remarked that it was still tolerable for multiple hours of use.

## Discussion

We developed a novel, universal 3D-printed socket that rapidly provides myoelectric control to individuals with transradial amputation in research settings. The 3D-printed fabrication approach has shown to be an appropriate short-term alternative to conventional techniques, offering high functionality at a small fraction of the time and cost of traditional socket-fabrication methods (Hallworth et al., 2020; Ismail et al., 2020). Our socket can be fabricated in ~7 h at any time prior to participant arrival, custom fit within 10 mins, and donned in under a minute. The total cost of materials of our functional-test socket is ~$8.00 USD. In contrast, traditional sockets, albeit intended for long-term use, require three to four visits with a prosthetist over 3–6 weeks and result in an estimated $800 to $3,000 USD before affiliated labor costs (Types of Prosthetics and Other Prosthetic FAQs Handspring; Frossard et al., 2017). Although less expensive diagnostic check sockets are routinely created by prosthetists during the socket fabrication process, they are intended to check proper fit rather than for functional use, much less myoelectric control (Quigley, 1985; Olsen et al., 2021). Our design can greatly increase participant involvement by further reducing the price per participant, making custom myoelectric control far more accessible.

Other attempts have been made to reduce costs and promote socket accessibility for individuals with upper-limb amputation. One group used photogrammetry to create a 3D scan of the residual limb onto which a socket could be generated and then fabricated using 3D printing (Ismail et al., 2020). This approach required at least two participant visits: one to scan the limb and another post-fabrication to test the socket. Additionally, the resulting socket was inherently specific to a single individual and would not generalize to others without repeating the fabrication process. Another group explored a low-cost and time-efficient solution for low- and middle-income countries that did not require electricity and instead used a rapidly expanding foam for socket adhesion (Miller et al., 2020). Though cost-effective ($100 USD) and quick to fabricate (~90 mins), this socket was specific to one individual, and a harness was needed to maintain a proper fit. Additionally, myoelectric control was not explored with this prosthesis. In another study, this same group also developed a gel-liner system with embedded electrodes that could be used with an individual's existing traditional transhumeral socket to enable myoelectric control (Reissman et al., 2018). Don times for the liner and the prosthesis were ~30 secs, and the liner was tested at home by the users over an average of 7 weeks. In that study, cost was not a specified design requirement, and, while not explicitly discussed, it can be reasonably assumed that it might be relatively high given the materials used and the customized nature of the device. Finally, a group explored a modular, low-cost myoelectric prosthetic platform that could accommodate individuals with or without transhumeral amputation and various terminal devices (Hallworth et al., 2020). While promising, it was only tested on able-bodied individuals, costs $200 USD, and performance was assessed *ad-hoc* in a gesture classification task rather than with a more functional, real-time task involving a physical prosthesis.

Our present work builds on these previous works and offers great customizability that addresses the high cost of traditional sockets as well as the variation in residual limbs, terminal devices, and DAQ methods. Our post-fabrication fitting process is especially useful for rapidly accommodating different residual limbs and individuals without requiring the reprinting of parts. Moreover, the socket adapts to residual-limb volume fluctuations and can be molded to avoid painful sites (e.g., bone protrusion, neuroma, etc.). Further, the adaptability to nearly any myoelectric terminal device and DAQ method indicates the value of this socket in a wide variety of research applications, potentially even beyond electromyography to applications in magnetomyography, sonomyography, or tactile myography (Kim and Colgate, 2012; Heidari et al., 2018; Calado et al., 2019; Dhawan et al., 2019; Connan et al., 2020). We note that in the present work, a single DAQ system was tested, and although additional DAQ systems for sEMG are theoretically compatible with the socket, these other systems have not been directly tested with this design. Other methods, could be potentially sensitive to the external pressure applied by the struts. The socket presented here can provide great value clinically for implementation with individuals interested in exploring the use of myoelectric prostheses; our socket allows for rapid use with no prior authorization from insurance nor financial commitment needed. The opportunity for individuals with transradial amputation to investigate myoelectric control with physical instead of virtual prostheses grants participants a more immersive and authentic experience to better determine if a myoelectric prosthesis is ideal for them. Importantly, the socket presented here is not intended to serve as a clinical diagnostic check socket, nor is it meant for long-term use as a final socket; rather, it is best utilized briefly in a research or clinical setting.

The functionality of the socket was not compromised in our design considerations to minimize cost and donning time and to promote adaptability. The mechanical testing demonstrated that, despite its low cost, the socket is robust under expected use conditions of at least 8 kg without detectable slipping or mechanical failure. We note that we did not test the socket to the point of mechanical failure in the present work but rather ensured reliability within use cases common in a research environment. Further testing would be necessary to ensure that the socket does not impose limitations for certain prostheses that are rated for loads beyond 8 kg (e.g., TASKA). We found that the socket presented here does not impede performance with the target-touching task and the BBT. Although neither test fully extrapolates to functionality with prosthesis use in ADLs, they both offer value in the investigation of performance with this socket as well as in comparison to traditional sockets (TASKA^*TM*^; i-Limb® Ultra; Roche et al., 2014; Alkhatib et al., 2019). It is interesting to note that performance during the target-touching task did not necessarily correlate with aptitude with the modified BBT. For example, P1 had a low mean PPT and high RMSE compared to other participants but fared equally well during the modified BBT. This is because the target-touching task requires precise control at ~50% flexion or extension in order to stay in target, which was not a position that the mKF was explicitly trained for. Thus, the decoding algorithm was required to generalize to a novel endpoint for both flexion and extension movements. On the other hand, the modified BBT is much more forgiving with the level of required contraction and could reasonably be accomplished by participants simply contracting beyond the level needed to make sufficient contact with a given block, without a penalty for grasping “too hard”. Additionally, the modified BBT does not test precision with extension movements and simply requires participants to relax in order to open the prosthesis sufficiently to drop each block. While we established that SNR as a group was not compromised by donning the socket, it is unclear why it varied considerably at the individual participant level. These differences could be attributed to changes in electrode adhesion to the residual limb with the added pressure from the socket. However, additional experiments would be needed to investigate why these discrepancies were more pronounced with SNR than with the other functional assessments. The comfort of our socket was acceptable throughout the entirety of the experiment, although it scored lower than participants' traditional sockets immediately following the fitting and donning process. For all participants, comfort degradation throughout their visit did not exceed the minimum detectable change, indicating adequate comfort for multiple hours of use.

Only three participants with specific levels of upper-limb amputation were recruited for this study. While our socket encountered no difficulties accommodating the variance in arm length or circumference across these three individuals, there is a great deal of variability in limb presentations; a larger group should be included in future studies to verify that the socket accommodates individuals who may present with significantly shorter residual limbs. Although the literature indicates that repeated heating and shaping of PLA will not degrade its mechanical properties (Chalgham et al., 2021), we did not methodically evaluate this claim with the present socket, and explicit tests would need to be performed to confirm this for our device.

Future work should focus on expanded implementation and improved comfort. Design and material considerations may accommodate other amputation levels (e.g., transhumeral or lower-limb) to further increase research participation. Greater comfort is of especial importance for the use of heavier terminal devices as to still allow for ADLs and functional tasks beyond controlling and lifting the prosthesis.

## Open-Source

The socket design has been open-sourced and is available for other researchers at https://github.com/utahneurorobotics/u-of-u-functional-test-socket. The repository contains .stl files for the 3D-printed parts and a parts list with links to other required off-the-shelf hardware. Additionally, an assembly video is provided in the repository.

## Data Availability Statement

The raw data supporting the conclusions of this article will be made available by the authors, without undue reservation.

## Ethics Statement

The studies involving human participants were reviewed and approved by the University of Utah Institutional Review Board (Protocol No. 98851). The patients/participants provided their written informed consent to participate in this study.

## Author Contributions

TH wrote the manuscript, designed experiments, recruited participants, and collected and analyzed the data. AC wrote the manuscript, investigated performance metrics, and collected and analyzed the data. ES refined the socket design by CB, collected the data, and assisted in drafting the manuscript. TT assisted in designing the experiments and collecting data. CB developed the initial version of the design and provided clinical support and expertise. CD provided clinical support and expertise throughout. JG oversaw the development of all aspects of the research. All authors contributed to the article and approved the submitted version.

## Funding

This research was supported by NIH 1 DP5 OD 029571-01.

## Conflict of Interest

CB is employed by Handspring. AC was a voluntary intern at Handspring. The remaining authors declare that the research was conducted in the absence of any commercial or financial relationships that could be construed as a potential conflict of interest.

## Publisher's Note

All claims expressed in this article are solely those of the authors and do not necessarily represent those of their affiliated organizations, or those of the publisher, the editors and the reviewers. Any product that may be evaluated in this article, or claim that may be made by its manufacturer, is not guaranteed or endorsed by the publisher.
